# Discovery of LAMP-2A as potential biomarkers for glioblastoma development by modulating apoptosis through N-CoR degradation

**DOI:** 10.1186/s12964-021-00729-8

**Published:** 2021-03-24

**Authors:** Yongjie Wang, Buyi Zhang, Jianli Wang, Haijian Wu, Shenbin Xu, Jianmin Zhang, Lin Wang

**Affiliations:** 1 Department of Neurosurgery, 2Nd Affiliated Hospital, School of Medicine, Zhejiang University, 88# Jiefang Road, Hangzhou, 310009 Zhejiang China; 2 Department of Pathology, 2Nd Affiliated Hospital, School of Medicine, Zhejiang University, 88# Jiefang Road, Hangzhou, 310009 Zhejiang China

**Keywords:** Glioblastoma, LAMP-2A, Chaperone-mediated autophagy, Nuclear receptor co-repressor, Unfolded protein response, Apoptosis

## Abstract

**Background:**

Lysosome-associated membrane protein type 2A (LAMP-2A) is the key component of chaperone-mediated autophagy (CMA), a cargo-selective lysosomal degradation pathway. Aberrant LAMP-2A expression and CMA activation have been demonstrated in various human malignancies. The study focusing on the intrinsic role of LAMP-2A and CMA in glioblastoma (GBM), and downstream mechanism could provide valuable insight into the pathogenesis and novel therapeutic modality of GBM.

**Methods:**

The levels of LAMP-2A, nuclear receptor co-repressor (N-CoR), unfolded protein response (UPR) and apoptosis were examined in clinical samples. LAMP-2A siRNA and shRNA were constructed to manipulate CMA activation. The role of CMA and downstream mechanism through degradation of N-CoR and arresting UPR mediated apoptosis were explored in GBM cells and nude mouse xenograft model.

**Results:**

Elevated LAMP-2A and associated decreased N-CoR expression were observed in GBM as compared with peritumoral region and low-grade glioma. Inhibited UPR and apoptosis were observed in GBM with high LAMP-2A expression. In vitro study demonstrated co-localization and interaction between LAMP-2A and N-CoR. LAMP-2A silencing up-regulated N-CoR and aroused UPR pathway, leading to apoptosis, while N-CoR silencing led to an opposite result. In vivo study further confirmed that LAMP-2A inhibition arrested tumor growth by promoting apoptosis.

**Conclusions:**

Our results demonstrated the central role of CMA in mediating N-CoR degradation and protecting GBM cells against UPR and apoptosis, and provided evidence of LAMP-2A as potential biomarker. Further research focusing on CMA with other tumorigenic process is needed and selective modulators of LAMP-2A remain to be investigated to provide a novel therapeutic strategy for GBM.

**Video Abstract**.

**Supplementary Information:**

The online version contains supplementary material available at 10.1186/s12964-021-00729-8.

## Background

Glioblastoma (GBM) is the most prevalent and aggressive primary brain tumor. Even with maximal safe resection, standard radiotherapy and temozolomide chemotherapy, the median survival is only 14.6 months [1]. Although advances in molecular profiling promote the identification of various potential biomarkers and treatment targets, including the famous monoclonal anti-VEGF-A antibody bevacizumab, there has not been significant improvement in survival of GBM [2, 3]. Therefore insights into the potential biomarkers and molecular mechanism for GBM development and progression still remain important in seeking new effective therapeutic targets.

As an evolutionarily conserved proteolytic pathway, autophagy maintains intracellular stasis through lysosome-mediated degradation [4]. The pathophysiological pathways of commonly known macroautophagy and microautophagy have been well described. Chaperone-mediated autophagy (CMA), on the contrary, is a highly selective degradation process targeting KFERQ-like-motif-bearing proteins, during which the substrates are recognized by heat shock cognate 71 kDa protein (Hsc70) and co-chaperones, and delivered to the surface of the lysosome where they bind to lysosomal associated membrane protein 2A (LAMP-2A) and undergo unfolding, and are finally translocated into the lysosomes where they are rapidly degraded [5–7]. The process is crucial for cellular homeostasis and adaptation to various forms of stress [8]. The specific characteristics of CMA lead to the researches that proved the linkage of deregulated CMA to the progression of various diseases [9]. Abnormally up-regulated LAMP-2A, as an indicator of activated CMA, has been demonstrated in a variety of human malignancies [10, 11]. Recent published papers identified the role of LAMP-2A in modulating GBM-pericytes interaction and temozolomide resistance via CMA [12–14]. However, the intrinsic function of LAMP-2A and CMA in GBM development and progression has been poorly elucidated.

Nuclear receptor co-repressor (N-CoR) is a vital component of a multi-protein repressor complex that is normally confined to nucleus and essential for the function of tumor suppressor proteins [15]. Misfolded N-CoR is transported out of nucleus and accumulated in the cytoplasm during malignant transformation of normal cells [16]. However, exceedingly intracytoplasmic aggregation of N-CoR activates unfolded protein response (UPR) and fires apoptosis cascade as means of self-destruction to stop canceration, via various stress sensing proteins anchored on endoplasmic reticulum (ER), including pancreatic ER kinase (PERK)-C/EBP homologous protein (CHOP) and inositol requiring enzyme 1 (IRE1)-JUN N-terminal kinase (JNK) pathway [16–18]. How cancer cells survive from stress caused by harmful protein accumulation remains unclear. In acute promyelocytic leukemia and non-small cell lung cancer, CMA is found to mediate misfolded N-CoR clearance to promote cancer cell survival [19, 20]. In GBM, although correlation between low level of N-CoR and tumor development and aggressiveness has been found [21, 22], the upstream regulatory mechanism is unknown.

Here we explored the expression of LAMP-2A in GBM and whether CMA sheltered tumor cells away from the accumulative stress from N-CoR. In this report, we first proved elevated LAMP-2A and decreased N-CoR expression in GBM clinical samples, and demonstrated that LAMP-2A targeted N-CoR for CMA degradation and as a result promoted the proliferation of GBM by inhibiting UPR mediated apoptosis in GBM cell and nude mice xenograft models. The findings provide support for LAMP-2A as potential biomarkers for GBM and valuable insight into the pathogenesis and novel therapeutic modality of GBM.

## Methods

### Clinical samples

A total of 24 tissue specimens were obtained from low grade glioma (LGG, WHO II, n = 8), tumor center (n = 8) and peri-tumor edema zone (PTEZ, n = 8) of GBM in the Second Affiliated Hospital of Zhejiang University School of Medicine from January 2017 to January 2018. Glioma was diagnosed according to the 2016 WHO Classification of Tumors of the Central Nervous System. Written informed consents were obtained from all the patients receiving surgical interventions. The study was approved by the Ethical Committee of the Second Affiliated Hospital of Zhejiang University School of Medicine. The clinical samples were used for quantitative polymerase chain reaction (qPCR), western blot (WB) and immunohistochemistry (IHC) analyses.

### Cell cultures and cell transfection

The glioma cell line U87-MG was purchased from the Cell Library of the Chinese Academy of Sciences (Shanghai, China). U87-MG was maintained in Dulbecco’s modified Eagle’s medium (DMEM; Gibco, Carlsbad, CA, USA) with 10% fetal bovine serum (FBS, Gibco) and 100 U/ml penicillin–streptomycin (Gibco). Starvation experiment was performed in DMEM medium without FBS for 24 h before being harvested for further analysis. For selection of efficient LAMP-2A and N-CoR siRNA, cells were cultured in 6-well plate at density of 5 × 10^5^/ml until confluence of 60–70%. The medium was replaced by Opti-MEM (Invitrogen, Grand Island, NY, USA). The following siRNAs were designed and transfected according to the Lipofectamine 2000 protocol (Lipo2000, Invitrogen): Negative control of LAMP-2A, 5′ → 3′-UUCUCCGAACGUGUCACGU; LAMP-2A siRNA1, 5′ → 3′-CUGGGAUGUUCUUGUACAA; LAMP-2A siRNA2, 5′ → 3′-GCUCUACUUAGACUCAAUA; LAMP-2A siRNA 3, 5′ → 3′-GCCUUGGGCUUCCUUAUAA; Negative control of N-CoR, 5′ → 3′-UUCUCCGAACGUGUCACGU; N-CoR siRNA1, 5′ → 3′-GACGAGUCAAGUUCAUUAA; N-CoR siRNA2, 5′ → 3′-GACCCUAUGAAAGUGUAUA; LAMP-2A siRNA 3, 5′ → 3′-CCUCGUCAGAAGGAAUUAU. The cells were harvested after 72 h post-transfection for mRNA quantification. The siRNA with best inhibitory effect was utilized for in vitro study and constructing LAMP-2A shRNA lentivirus. To study the involvement of apoptosis, U87-MG cells were pretreated with pan-caspase inhibitor ZvaD (MedChemExpress, NJ, USA) at concentration of 40 μm for 30 min before being transfected with siRNAs.

### Constructing lentivirus-mediated LAMP-2A shRNA

The pLKO.1-shLAMP-2A and control plasmid were designed and cloned, together with package plasmids including psPAX2 and PMD2G. These lentivirus-based constructs were co-transfected into cultured 293 T cells. The replication-deficient lentiviral particles were packaged and collected 72 h later for in vivo study.

### Nude mouse xenograft studies

The study was approved by the Institutional Animal Care and Use Committee of The Second Affiliated Hospital of Zhejiang University, School of Medicine. The male BALB/c nude mice (average weight 20 g; 6–8 weeks old) were purchased from Experimental Animal Laboratories (SLAC, Shanghai, China). The mice were cultivated in a specific pathogen-free room at 20 °C under a 14 h light/10 h dark cycle with free access to food and water. Nine mice were randomly subdivided into three groups: vehicle group was injected with normal U87-MG cells; negative control group was injected with U87-MG cells transfected with control virus; LAMP-2A shRNA group was injected with U87-MG cells transfected with LMAP-2A shRNA virus. The allocation process was blinded to the researcher. Mice were injected subcutaneously under the axillary with treated U87-MG cells at a density of 1 × 10^7^ and the tumor volume (mm^3^ = length × width × height × 0.5236) were calculated from day 12 until day 30. All mice were sacrificed by intraperitoneal injection of 100 mg/kg of barbiturate and the tumor tissues were harvested as a whole to obtain weight before being cryopreserved for further analysis.

### Quantitative real-time RT-PCR

Total RNA from cultured cells was extracted using TRIzol reagent (Invitrogen) according to the manufacturer’s instructions. cDNA was generated by 1 μg RNA using the RevertAid First Strand cDNA Synthesis Kit (MBI Fermentas, Burlington, Canada).

Quantitative real-time RT-PCR (qRT-PCR) was carried out with the Power SYBR Green Master Mix (Thermo Fisher Scientific, Wilmington, DE, USA) on ABI 7300 real-time quantitative PCR system (Life Technologies, Grand Island, NY) with the following conditions: 95 °C for 10 min, 40 cycles of 95 °C for 15 s, 60 °C for 1 min.The specific primers were list as follows: LAMP-2A, forward 5′CAATAGCAGCACCATTAAG3′, reverse 5′GGAGCCATTAACCAAATAC3; N-CoR, forward 5′AGGCGACACAATCTTGAC3′, reverse 5′ TGGAAGCGACACTTTCAC 3′; β-actin as internal reference, forward 5′ CATCGTCCACCGCAAATGCTTC 3′, reverse 5′ AACCGACTGCTGTCACCTTCAC 3′. Results were analyzed using the 2^−∆∆C^ method.

### Western blot

After various treatment, brain tissues or cell lysates were prepared with immunoprecipitation assay (RIPA) lysis buffer (solarbio, Dalian, China). BCA kit (Thermo Fisher Scientific, Wilmington, DE, USA) was applied to measure the protein concentrations. The lysates containing 40 μg of protein were subjected to SDS-PAGE and transferred to nitrocellulose filter membrane (Millipore, Bedford, MA, USA). After blocking with 10% bovine serum albumin at room temperature for 1 h, the membranes were incubated overnight at 4 °C with primary antibodies as follows: LAMP-2A(1:1000, Abcam, Cambridge, UK, ab125068), N-CoR (1:500, Abcam, a ab58396), *p*-PERK (1:300, Santa Cruz, CA, USA, discontinued product, sc-32577) (1:1000, Cell Signaling Technology, Beverly, MA, #3179S), PERK (1:1000, CST, #5683S), *p*-IRE1 (1:2000, Abcam, ab124945), IRE1 (1:2000, Abcam, ab37073), *p*-JNK (1:1000, Abcam, ab124956), JNK (1:1000, Abcam, ab179461), CHOP (1:1000, Abcam, ab11419), Bcl-2 (1:300, Santa Cruz,sc-492), Bax (1:300, Santa Cruz, sc-493), Caspase-3(1:500, Abcam, ab44976), Caspase-9 (1:1000, Abcam, ab2013), GAPDH (1:2000, CST, #5174), Hsc70(1:3000, Proteintech, IL, USA, 66,442–1-Ig), Lamin B1(1:1000, Abcam, ab16048) and β-actin(1:1000, CST, #4970). Then the membranes were probed with horseradish peroxidase labeled secondary antibody (goat anti-rabbit, donkey anti-goat, goat anti-mouse, 1:1000, Beyotime, Shanghai, China), and visualized with chemiluminescence). NE-PER™ kit (Thermo Fisher Scientific) was used for nuclear and cytoplasmic protein extraction. The bands were quantified by Image software (ImageJ) from National Institutes of Health (http://rsb.info.nih.gov/ij/download.html).

### Immunohistochemistry staining

Immunohistochemistry was performed with LAMP-2A (1:100, Abcam, ab18528), N-CoR (1:100, Santa Cruz, sc-1609) for cells and N-CoR (1:100, Novus, CO, US, NBP1-28,863) for tissues. After being equilibrated at − 20 °C for 15 min, brain tumor tissues were sectioned at a range of 5–7 μm and fixed with 4 °C pre-cold acetone. Cultured cells growing on glass slides were fixed with 4% of formaldehyde. The tissue or cell sections were incubated with 3% of H_2_O_2_ for 10 min, followed by 10% of BSA as blocking solution for 1 h at room temperature. Then the sections were incubated with the primary antibodies overnight at 4 °C and hybridized with horseradish peroxidase labeled secondary antibody (Beyotime, China) for 1 h at room temperature. The diaminobenzidine kit (DAB kit; Long Island Biotec, Shanghai, China) was applied for visualization and hematoxylin (BASO, China) for counterstain.

### Immunofluorescence staining

The brain tissue and cell sections were prepared. After permeabilization with 0.3% Triton X-100 (Sigma-Aldrich), sections were probed with LAMP-2A (1:100, Abcam, ab18528), N-CoR (1:100, Novus, NBP1-28863) for tissues or N-CoR (1:100, Santa Cruz, sc-1609) for cells. After being washed with PBS containing 0.05% of Tween-20 (Sigma-Aldrich), sections were incubated with fluorescent secondary antibodies (Beyotime, China) and antifade mounting medium containing 1:500 DAPI (Beyotime, China). Leica fluorescent microscope was used for visualization.

### Co-immunoprecipitation (Co-IP)

Cultured U87-MG cells were lysed in lysis buffer (20 mM pH 7.5 Tris, 150 mM NaCl, 1% TritonX-100, 1 mM EDTA and protease inhibitor cocktail). The lysates were cleared by centrifugation and subsequently incubated with either 20 μg of IgG or 2 μg of LAMP-2A antibody(Abcam, 125068), or 2 μg of Hsc70 antibody(Proteintech, 10654-1-AP) for 2 h with rotation on ice. Protein G-Agarose beads (Roche, Mannheim, Germany) were then added followed by overnight rotation at 4 °C. Immunoprecipitants were separated on SDS-PAGE gel. LAMP-2A(1:1000, Abcam, 125068), Hsc70 (1:3000, Proteintech, 66442-1-Ig) and N-CoR (1:500, Abcam, ab3482) were detected by western blotting as previously mentioned.

### Terminal deoxynucleotidyl transfer-mediated dUTP nick end labeling (TUNEL)

Cultured cells growing on glass slides were fixed with 4% of formaldehyde. After permeabilization with 0.3% Triton X-100 (Sigma-Aldrich), apoptotic cells were detected according to the manufacturer’s protocol of TUNEL kit (Roche).

### Flow cytometry detection of apoptosis

Cultured cells growing on 6-well plate with various treatment were digested. Apoptosis was measured by the Annexin V-fluorescein isothiocyanate apoptosis kit (Beyotime, China) according to the manufacturer’s protocol. The results were analyzed with a FACS Calibur flow cytometer (BD Biosciences).

### Statistical analysis

Numerical data were expressed as means ± standard error of mean (SEM) from at least 3 independent experiments. The statistical analyses were performed by One-Way ANOVA and Bonferroni's multiple comparisons test. All statistical analyses were performed with SPSS 18.0 software (SPSS, Inc., Chicago, IL), and *p* < 0.05 was considered to be statistically significant.

## Results

### Expression of LAMP-2A in different regions of GBM and LGG

**Fig. 1:**
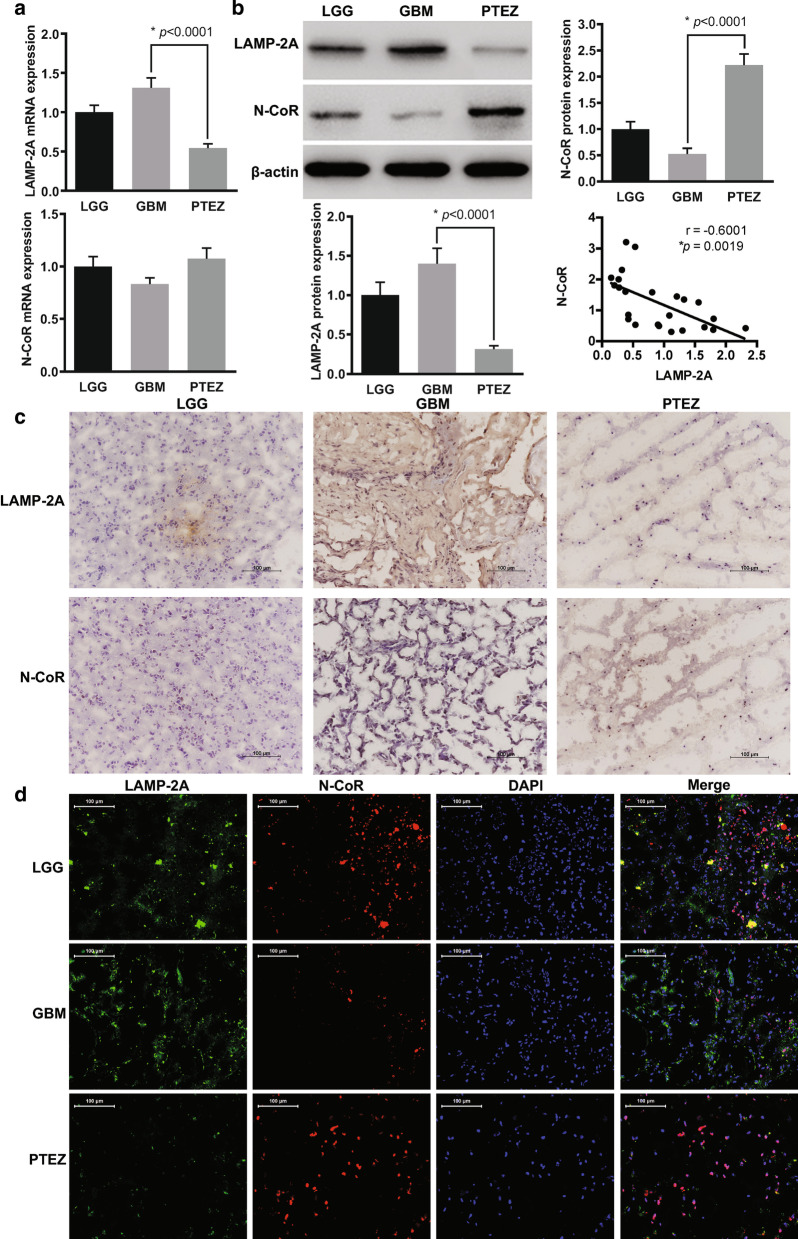
Expressive analysis of LAMP-2A and N-CoR in clinical samples. **a** mRNA levels of LAMP-2A and N-CoR were measured by qRT-PCR. **b** Protein levels of LAMP-2A and N-CoR were measured by western blot. LAMP-2A mRNA and protein levels were significantly increased in GBM center (n = 8) in comparison with peri-tumor edema zone (PTEZ, n = 8) (*p* < 0.0001), while increasing trend was observed as compared with low grade glioma (LGG, n = 8). The protein level of N-CoR, but not mRNA level was significantly decreased in GBM center as compared with PTEZ (*p* < 0.0001). Linear regression analysis incorporating data from LGG, GBM center and PTEZ revealed moderate negative correlation between protein expression of LAMP-2A and that of N-CoR (r =  − 0.6001, *p* = 0.0019). **c** Immunohistochemistry (IHC) analysis of LAMP-2A and N-CoR (brown signal) in glioma clinical samples. Nucleus (blue signal) was stained with hematoxylin; D. immunofluorescence (IF) analysis of LAMP-2A (green signal) and N-CoR (red signal) in glioma clinical samples. DNA (blue signal) was stained with DAPI. Both IHC and IF studies displayed upregulation of LAMP-2A and downregulation of N-CoR in GBM centers. The data are mean ± SEM from 8 tissue specimens as a group. mRNA or protein levels are expressed relative to LGG set as 1. Significant changes are set as *p* < 0.05 and represented by asterisk (One-Way ANOVA; Bonferroni's test)

To determine the expressive profile of LAMP-2A in clinical samples, we analyzed mRNA by qRT-PCR, protein by western blot (WB), in-situ tissue expression by immunohistochemistry (IHC) and immunofluorescence (IF) in GBM center, PTEZ of GBM and LGG. Increased trend and significant increased expression of LAMP-2A at both mRNA and protein level were observed in GBM center compared with LGG and PTEZ relatively (Fig. 1a, b). Both IHC and IF analysis displayed upregulation of LAMP-2A in GBM center (Fig. 1c, d). The elevated expression of LAMP2A suggested the hypothesis that CMA might be playing a role in tumor formation.

### Post-transcriptional loss of N-CoR in GBM was correlated with LAMP-2A expression

The protein level of N-CoR, but not mRNA level was markedly decreased in GBM center as compared with PTEZ, as indicated by WB and qRT-PCR (Fig. 1a, b), implying post-transcriptional modification. Further linear regression analysis from protein expression profile of LGG, tumor center and PTEZ revealed moderate negative correlation between expression of LAMP-2A and that of N-CoR at protein level(r =  − 0.6001, *p* = 0.0019). Both IHC and IF analysis confirmed the inverse association between LAMP-2A and N-CoR (Fig. 1c, d). These findings prompted us to explore the interaction between LAMP-2A and N-CoR, and the downstream pathway.

### Activation of UPR and caspase pathway in GBM with different LAMP-2A levels

**Fig. 2:**
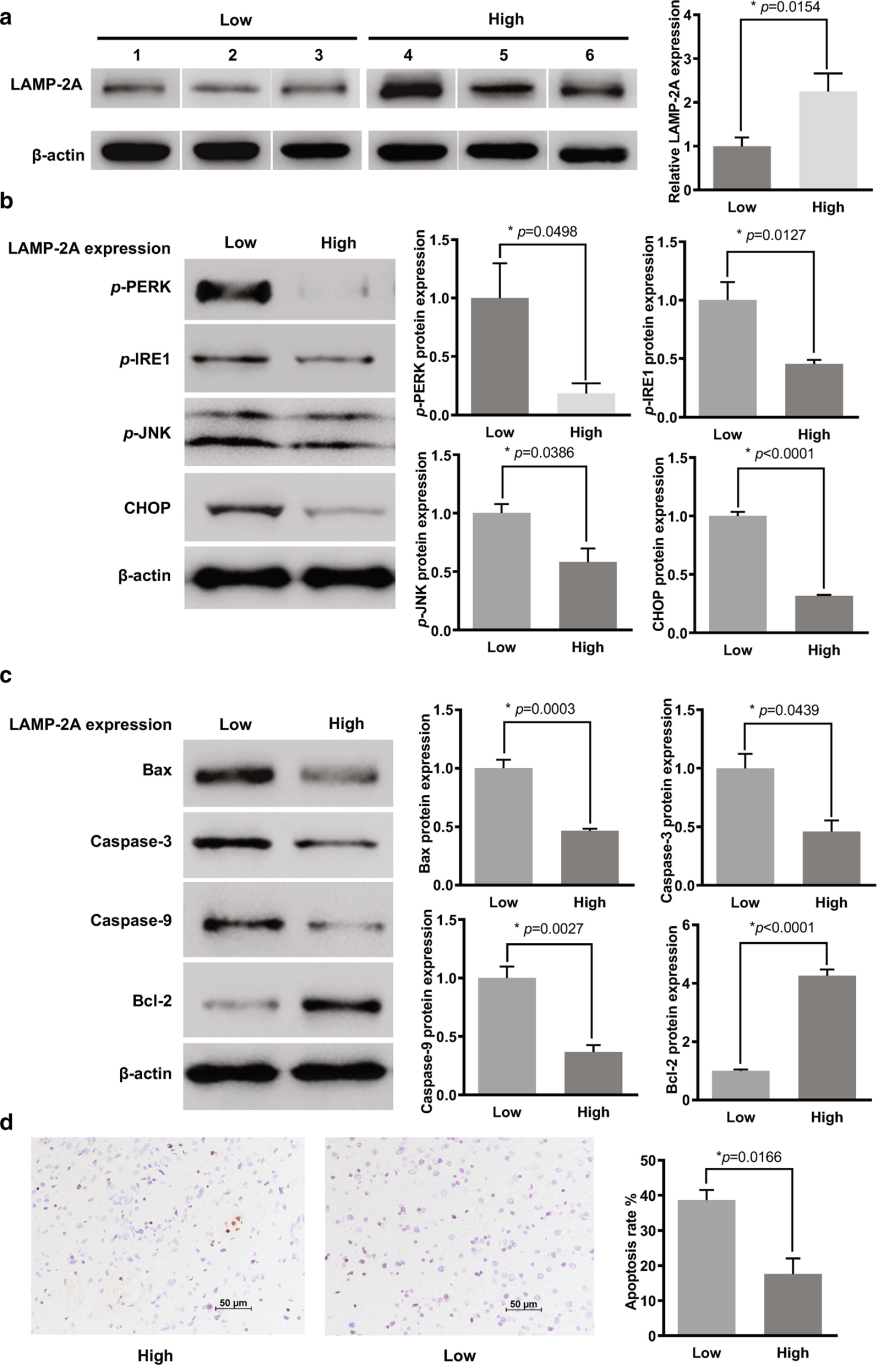
Analysis of activation of UPR and caspase pathway and UPR in GBM samples with different LAMP-2A expression. **a** We divided six GBM samples according to the LAMP-2A level as “High” expression group and “Low” expression group. The average LAMP-2A expression of the High group was about two times of that of the Low group. **b** UPR activation was examined by protein expression of *p*-PERK, *p*-IRE1, *p*-JNK and CHOP. Significant down-regulation of *p*-PERK, *p*-IRE1, *p*-JNK and CHOP was observed in the High group as compared with the Low group. **c** Activator proteins in apoptosis including caspase 3, caspase 9 and Bax, and anti-apoptotic protein Bcl-2 were measured by western blot. Caspase 3, caspase 9 and Bax expressions were inhibited while Bcl-2 expression was enhanced in the High group as compared with the Low group. **d** Apoptotic levels were examined by Tunnel analysis and compared within the two groups, with positive cells stained by orange. Patients with high LAMP-2A expression showed as half apoptosis rate as those with low LAMP-2A expression. The data are mean ± SEM from three glioblastoma samples. Protein levels are expressed relative to Low group set as 1. Significant changes are set as *p* < 0.05 and represented by asterisk (Two-tailed t test)

GBMs were divided into two groups according to LAMP-2A level for further analysis of UPR and caspase pathway activation. Three patients with low LAMP-2A protein level were allocated to the “Low” group, and the other three patients with high LAMP-2A protein level to the “High” group. The average LAMP-2A expression in the High group was about twice as much as that in the Low group (Fig. 2a). Significant down-regulation of key components of UPR pathway, namely *p*-PERK, *p*-IRE1, *p*-JNK and CHOP, was detected in the High group compared with Low group (Fig. 2b). The apoptotic level in the High group was significantly lower than that in the Low group, as indicated by down-regulation of caspase 3, caspase 9 and Bax (pro-apoptotic protein), and up-regulation of Bcl-2 (anti-apoptotic protein) (Fig. 2c). TUNEL analysis revealed about half apoptotic rate in the High group than that in the Low group (Fig. 2d). The above findings in clinical samples led to the hypothesis that UPR and apoptotic pathway might be the downstream effector of CMA.

### Effect of LAMP-2A knockdown on CMA substrate

**Fig. 3:**
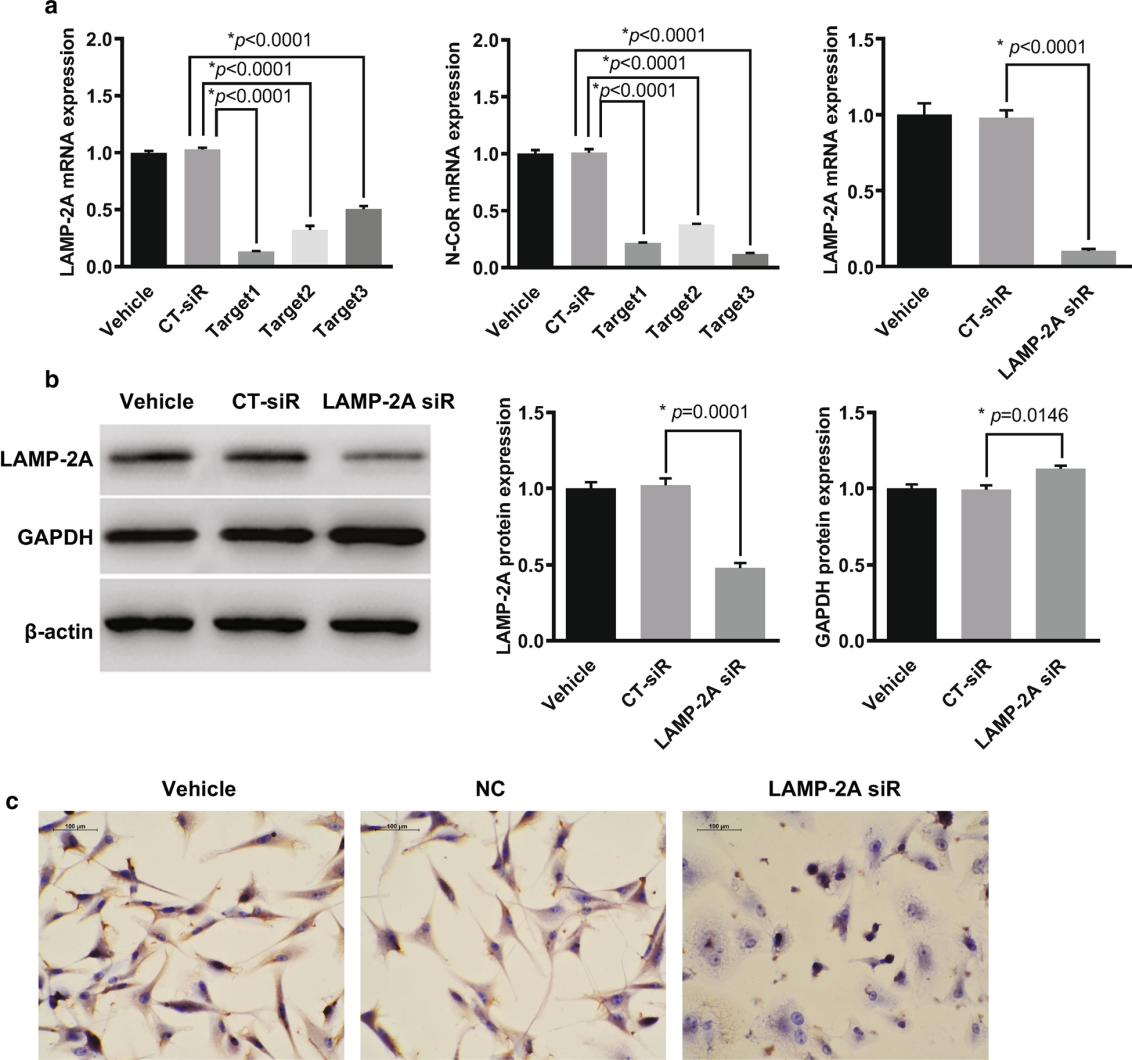
Verification of LAMP-2A and N-CoR siRNA, LAMP-2A shRNA plasmid efficiency, and the effect of LAMP-2A expression on CMA substrate. **a** The suppressive effects of target siRNAs for LMAP-2A (left), target siRNAs for N-CoR (middle) and shRNA plasmid for LAMP-2A (right) were examined in U87-MG cells by qRT-PCR. LAMP-2A siRNA1 and N-CoR siRNA3 showed highest suppressive efficiency in comparison with control siRNA (CT-siR) and were selected for further experiments. shRNA plasmid was designed based on LAMP-2A siRNA (LAMP-2A siR) which showed high inhibitory function on LAMP-2A mRNA expression as compared with control shRNA (CT-shR). **b** Western blot demonstrated inhibitory efficiency of LAMP-2A siR1 in contrast with CT-siR in U87-MG cells. GAPDH was slightly but significantly increased in the U87-MG cells when LAMP-2A was silenced. **c** Immunohistochemistry analysis confirmed the knockdown effect of LAMP-2A siR on LAMP-2A expression (brown signal) in contrast with CT-siR in U87-MG cells. Nucleus (blue signal) was stained with hematoxylin. The data are mean ± SEM from three independent experiments. Protein and mRNA levels are expressed relative to vehicle treatment group set as 1. Significant changes are set as *p* < 0.05 and represented by asterisk (One-Way ANOVA; Bonferroni's test)

The basal level of LAMP-2A was high in U87-MG cells (Fig. 3b, c). To explore the downstream effect of LAMP-2A inhibition, we constructed three target siRNAs, among which LAMP-2A siRNA1 showed highest suppressive efficiency on qRT-PCR (13.2%, *p* < 0.0001) and was selected for the following experiments (Fig. 3a). Further WB and IHC confirmed the knockdown effect of LAMP-2A siRNA in contrast with the control siRNA (CT-siRNA) (Fig. 3b, c). CMA can be monitored indirectly by investigating its targeted proteins such as glyceraldehyde-3-phosphate dehydrogenase (GAPDH), which contains a KFERQ motif and is typically not regulated by transcriptional mechanisms [23]. The expression of GAPDH was slightly but significantly increased in U87-MG cells when LAMP-2A was silenced (Fig. 3b).

### N-CoR interacted with LAMP-2A and was degraded by CMA in GBM cells

**Fig. 4:**
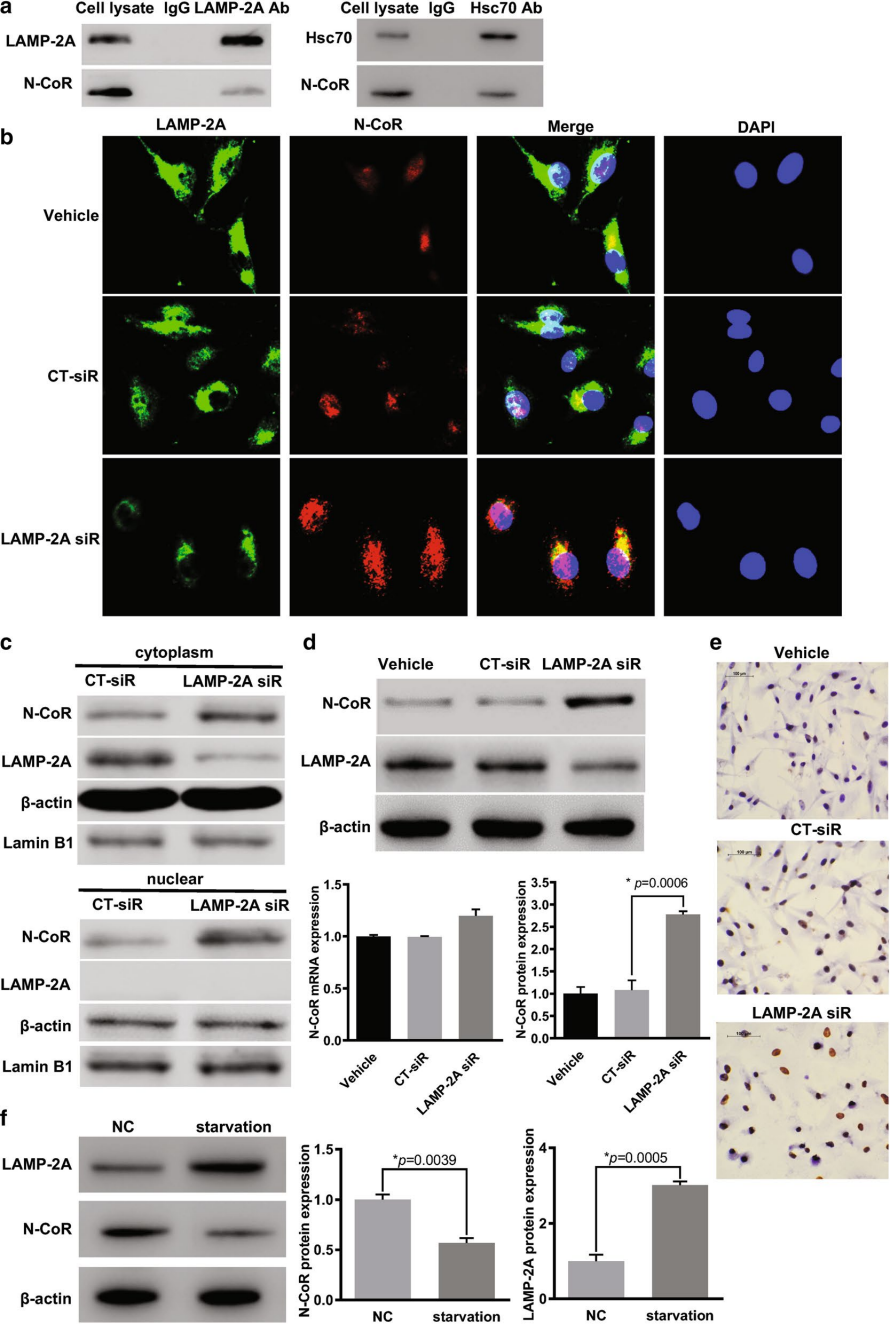
N-CoR was a substrate of chaperone mediated autophagy and modulated by LAMP-2A siRNA. **a** The association between N-CoR and LAMP-2A (left), N-CoR and Hsc70 (right) was confirmed by co-immunoprecipitation assay in U87-MG cells. The cell lysates immunoprecipitated by either LAMP-2A antibody or Hsc70 displayed positive N-CoR protein expression as detected by N-CoR antibody. **b** Distribution of LAMP-2A (green) and N-CoR (red) in U87-MG cells was demonstrated in immunofluorescence analysis. DNA (blue signal) was stained with DAPI. Significant co-localization between LAMP-2A and N-CoR was observed in vehicle and control siRNA (CT-siR) groups. After treatment with LAMP-2A siRNA (LAMP-2A siR), remarkable cytoplasmic and intra-nucleus accumulation of N-CoR was observed as compared with vehicle and CT-siR groups. **c** Nuclear and cytoplasmic proteins were separated by NE-PER™ kit. WB analysis showed that cytoplasmic LAMP-2A expression was inhibited, and N-CoR in both cytoplasmic and nuclear compartments were accumulated upon treatment with LAMP-2A siRNA as compared with CT-siR treatment. **d** the protein expression of N-CoR was significantly increased after LAMP-2A knockdown as compared with CT-siR in U87-MG cells, while the mRNA level of N-CoR remained unchanged. **e** Immunohistochemistry analysis confirmed increased protein level of N-CoR (brown signal) after LAMP-2A was inhibited in U87-MG cells. Nucleus (blue signal) was stained with hematoxylin. **f** Compared with normal control (NC), starvation overnight resulted in activation of CMA pathway, as demonstrated by increased protein expression of LAMP-2A, and significantly decreased N-CoR level. The data are mean ± SEM from three independent experiments. Protein and mRNA levels are expressed relative to vehicle treatment group set as 1. Significant changes are set as *p* < 0.05 and represented by asterisk (One-Way ANOVA; Bonferroni's test)

It has been reported in non-small cell lung cancer that N-CoR contains QEIFR pentapeptide and is the target of CMA [20]. Therefore, to test the direct association between N-CoR and LAMP-2A in GBM, together with Hsc70, another important component of CMA process which is responsible for targeting KFERQ-like-motif-bearing proteins, co-immunoprecipitation assay was performed. The cell extract was immunoprecipitated with anti-LAMP-2A and anti-Hsc70 respectively, and the interaction with either LAMP-2A and Hsc-70 was confirmed by WB with anti-N-CoR (Fig. 4a). Moreover, from indirect immunofluorescence assay, significant co-localization between LAMP-2A and N-CoR was observed in vehicle and CT-siRNA treated U87-MG cells (Fig. 4b). We further examined the effect of LAMP-2A ablation on the expression and distribution of N-CoR. Treatment with LAMP-2A siRNA reversed N-CoR loss at protein level, but not mRNA level (Fig. 4d, e). Upon activation of CMA by starvation, LAMP-2A expression dramatically increased, while N-CoR expression decreased accordingly, as indicated by WB analysis (Fig. 4f). In immunofluorescence assay (Fig. 4b), upon siRNA mediated knockdown of cytoplasmic LAMP-2A, CMA mediated N-CoR ablation in cytoplasm compartment was inhibited, and as a result remarkable cytoplasmic and intra-nucleus accumulation of N-CoR were observed. Since the remaining LAMP-2A also resided in the cytoplasm, it seemed that colocalization of both proteins increased. Furthermore, subcellular fractionation was conducted to separate nuclear and cytoplasmic proteins in CT-siRNA and LAMP-2A siRNA transfected cells, WB analysis confirmed knockdown of cytoplasmic LAMP-2A and N-CoR accumulation in both cytoplasmic and nuclear compartments upon treatment with LAMP-2A siRNA (Fig. 4c). These results proved that N-CoR was degraded by CMA through recognition by Hsc70 and interaction with LAMP-2A and explained the inverse relationship between LAMP-2A and N-CoR observed in clinical samples.

### LAMP-2A knockdown enhanced apoptosis in GBM cells

**Fig. 5:**
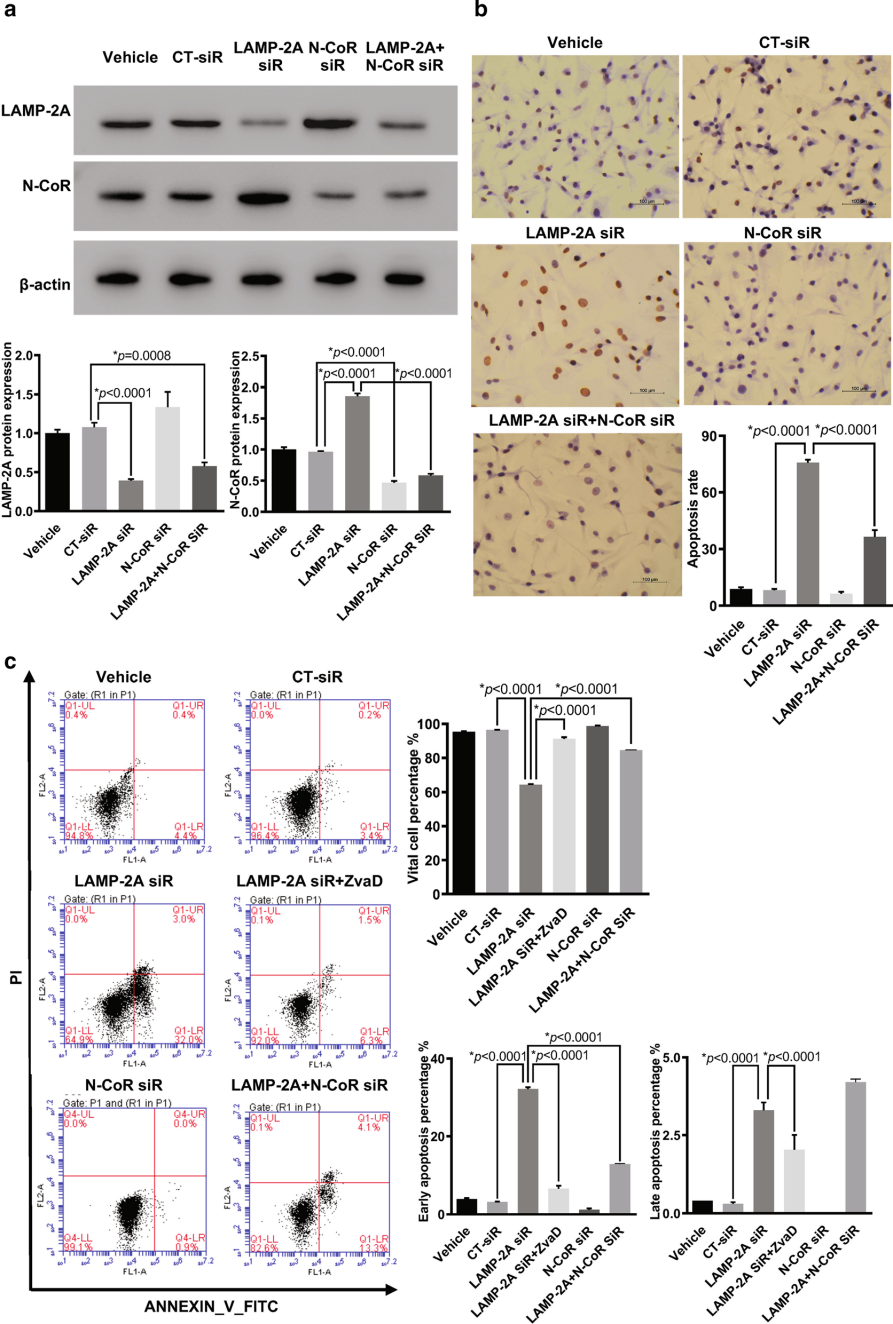
Effect of LAMP-2A and N-CoR expression on apoptosis in U87-MG cells. **a** Western blot analysis of LAMP-2A and N-CoR under different siRNA treatment conditions confirmed inhibitory effect of LAMP-2A siRNA (LAMP-2A siR) and N-CoR siRNA (N-CoR siR). Although LAMP-2A inhibition resulted in significant increase of N-CoR, addition of N-CoR siR almost eliminated this effect. **b** TUNEL results of differently treated U87-MG cells. LAMP-2A silence by siRNA significantly enhanced the apoptotic level when compared with control siRNA (CT-siR) transfections, while addition of N-CoR siR truncated the pro-apoptotic effect of LAMP-2A silence by half. **c** Flow cytometry analysis of differently treated U87-MG cells. Both early (right lower quadrant) and late apoptosis (right upper quadrant) levels were elevated when LAMP-2A was downregulated by siRNA. Again N-CoR ablation by siRNA eliminated enhanced apoptosis levels of LAMP-2A siR group by more than half. Treatment with ZvaD as pan-caspase inhibitor also reversed the pro-apoptotic effect of LAMP-2A ablation, confirming the involvement of caspase pathway as downstream effectors. The data are mean ± SEM from three independent experiments. Significant changes are set as *p* < 0.05 and represented by asterisk (One-Way ANOVA; Bonferroni's test)

First we proved the siRNA mediated extinction of LAMP-2A and N-CoR protein expression by western blot (Fig. 5a). The apoptosis rate of U87-MG cells with suppressed LAMP-2A was remarkably enhanced when compared with control transfections, as displayed by TUNEL analysis (Fig. 5b). Additionally, flow cytometry showed both early and late apoptosis levels were elevated when LAMP-2A was silenced (Fig. 5c). Moreover, the pretreatment with ZvaD, a pan-caspase inhibitor, resulted in the reversal of the pro-apoptotic effect of LAMP-2A silencing (Fig. 5c). These results supported the important role of LAMP-2A and CMA in improving GBM cell survival and promoting tumorigenesis, and the involvement of caspase pathway and apoptotic death in this process.

### N-CoR knockdown reversed the pro-apoptosis function of LAMP-2A siRNA

To prove whether CMA protected GBM cell from apoptosis through degradation of N-CoR, we designed and selected N-CoR targeted siRNA with highest suppressive efficiency (Fig. 3a). Upon N-CoR knockdown (Fig. 5a), the enhanced apoptosis resulting from LAMP-2A ablation was truncated by half, as demonstrated by TUNEL and flow cytometry (Fig. 5b, c). The results suggested that GBM might evade from apoptosis via CMA mediated N-CoR clearance.

### CMA inhibition resulted in activation of UPR and caspase pathway in N-CoR dependent manner

**Fig. 6:**
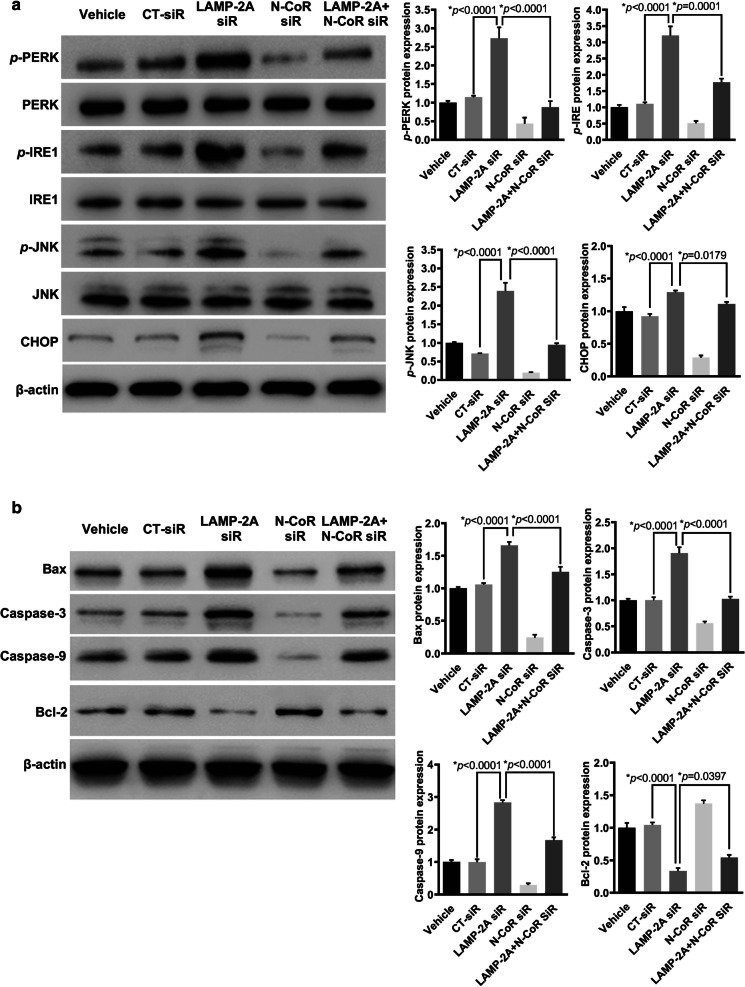
Effect of LAMP-2A and N-CoR expression on UPR activation and the apoptosis pathway in U87-MG cells. **a** UPR activation was examined by protein expression of p-PERK, p-IRE1, p-JNK and CHOP. LAMP-2A ablation resulted in remarkably increased expression of *p*-PERK, *p*-IRE1, *p*-JNK and CHOP, while N-CoR knockdown achieved opposite effect. These data together with findings from clinical samples, implied that CMA activation played important role in glioblastoma survival by degrading N-CoR and inhibiting downstream UPR activation. **b** Activator proteins in apoptosis including caspase 3, caspase 9 and Bax, and anti-apoptotic protein Bcl-2 were measured by western blot in differently treated U87-MG cells. Significant up-regulation of caspase 3, caspase 9 and Bax with simultaneous down-regulation of Bcl-2 were observed when LAMP-2A was inhibited, and these pro-apoptotic effects were reversed by N-CoR knockdown. The data are mean ± SEM from three independent experiments. The levels of *p*-PERK, *p*-IRE1 and *p*-JNK are compared with levels of PERK, IRE1 and JNK respectively, while the rest of the proteins are compared with β-actin. Protein levels are expressed relative to vehicle treatment group set as 1. Significant changes are set as *p* < 0.05 and represented by asterisk (One-Way ANOVA; Bonferroni's test)

To test whether the protective effect of CMA on GBM cells was linked to neutralization of UPR, a stress reaction caused by intracellular accumulation of N-CoR, we further examined PERK/CHOP and IRE1/JNK pathway, which have been proved to fire apoptosis cascade [16–18]. The silencing efficiency of LAMP-2A siRNA and N-CoR siRNA in U87-MG cells, alone or in combination, was proved as in Fig. 5a. LAMP-2A ablation resulted in remarkably increased expression of *p*-PERK, *p*-IRE1, *p*-JNK and CHOP (Fig. 6a). We further investigated the key proteins in the apoptosis pathway, and found significant up-regulation of caspase 3, caspase 9 and Bax with simultaneous down-regulation of Bcl-2 upon LAMP-2A inhibition (Figaure 6b). Furthermore, we examined the influence of N-CoR expression on the activated UPR and caspase pathway triggered by LAMP-2A ablation, and found that N-CoR knockdown achieved opposite effect, presented as decreased expression of *p*-PERK, *p*-IRE1, *p*-JNK, CHOP and apoptogenic proteins, and up regulation of anti-apoptotic protein, either by comparing with CT-siRNA group or LAMP-2A siRNA group (Fig. 6a, b). These findings collectively suggested that degradation of N-CoR by CMA might protect GBM cells against UPR resulting from excessive N-CoR accumulation and eventual avoid the activation of caspase pathway.

### CMA inhibition stunted the growth of glioma by restoring UPR-mediated apoptosis

**Fig. 7:**
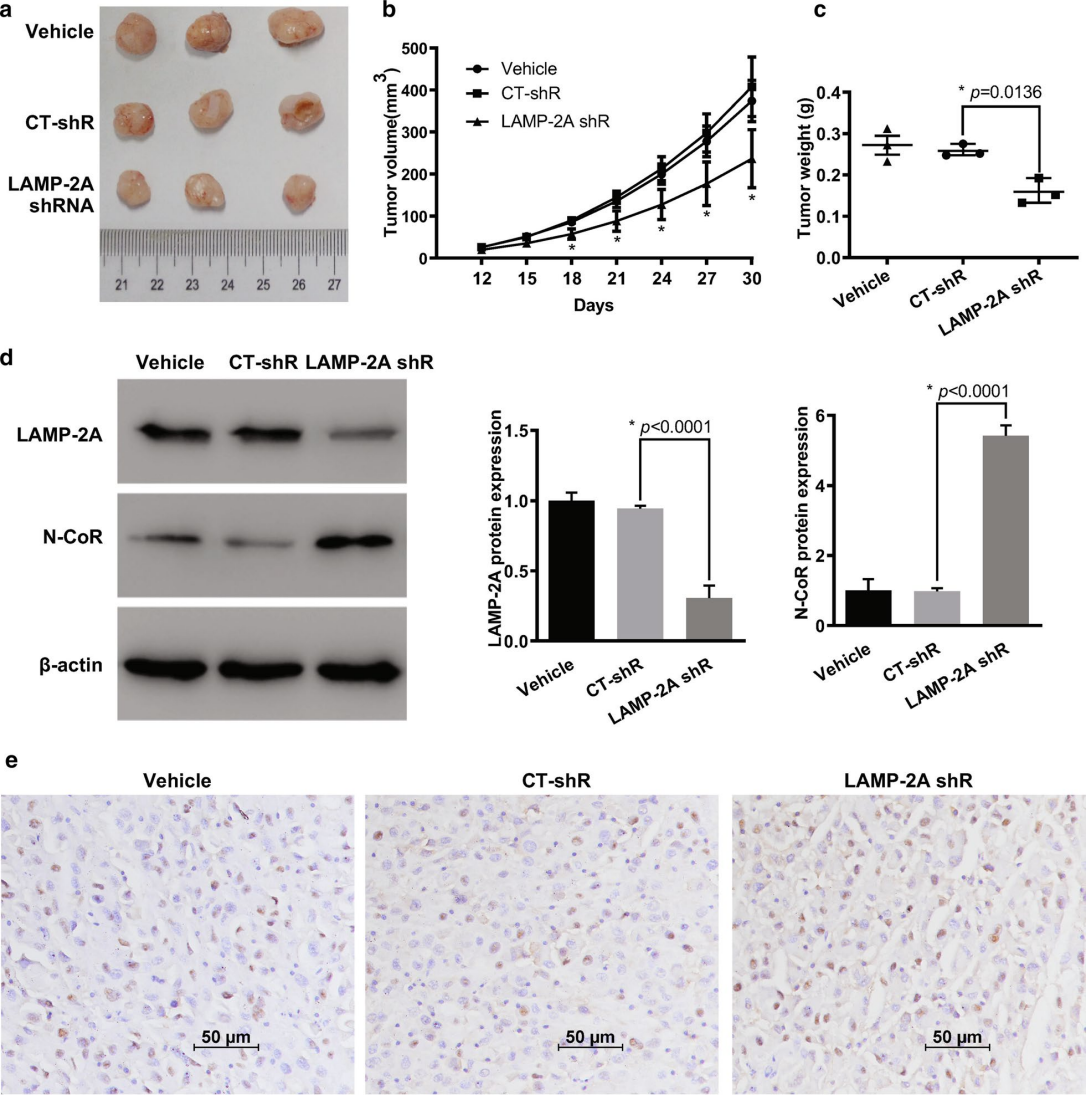
CMA blockage promoted N-CoR accumulation and suppressed tumorigenesis of glioma in vivo. **a** Representative image of xenograft tumors grown in nude mice injected with differently treated U87-MG cells. LAMP-2A shRNA (LAMP-2A shR) group showed significantly smaller tumors than those in control shRNA (CT-shR) group. **b** Continuous measurements of tumor volumes. The difference of tumor volumes between LAMP-2A shR and CT-shR group on Day 12 and 15 was insignificant. From post-implantation Day 18 till last measurement, the tumor volumes were significantly decreased in LAMP-2A shR group. **c** The final tumor weight after mice sacrifice was significantly lower in LAMP-2A shRNA group as compared with CT-shR group; D. Western blot analysis of LAMP-2A and N-CoR expression from tumor tissues of differently treated mice. Decreased LAMP-2A and increased N-CoR expression were observed in LAMP-2A shR group compared with CT-shR group; E. Immunohistochemistry analysis of N-CoR (brown signal) from tumor tissues of differently treated mice. Increased N-CoR expression was observed in LAMP-2A shR group compared with CT-shR group. The results were in accordance with the findings from clinical samples and in vitro study. The data are mean ± SEM from three mice as a group. Significant changes are set as *p* < 0.05 and represented by asterisk (One-Way ANOVA; Bonferroni's test)

**Fig. 8:**
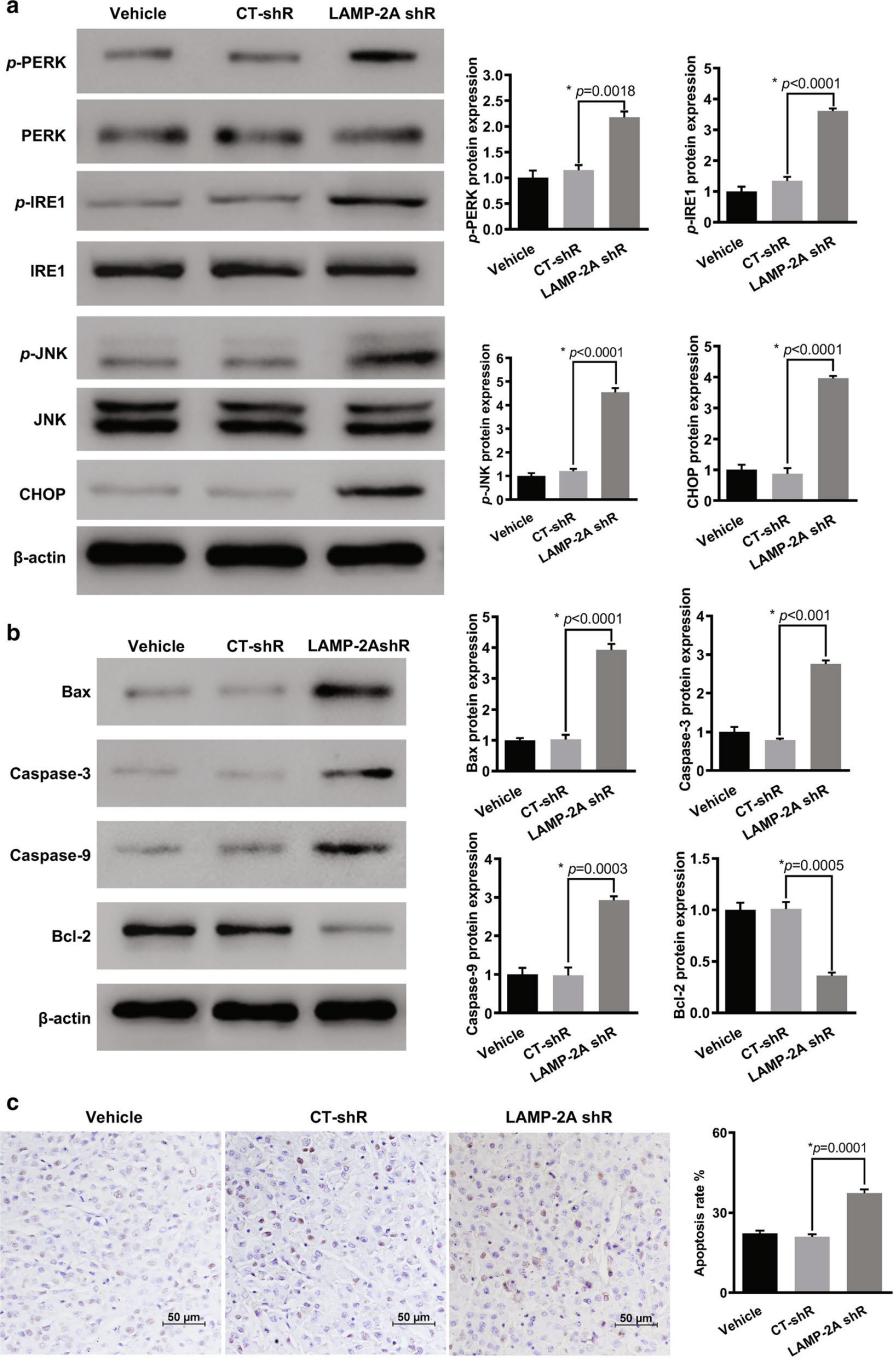
Effect of CMA blockage on downstream UPR and apoptosis pathway in a xenograft mouse model. **a** UPR activation was examined by protein expression of *p*-PERK, *p*-IRE1, *p*-JNK and CHOP. Significant up-regulation of *p*-PERK, *p*-IRE1, *p*-JNK and CHOP was observed in the LAMP-2A shRNA (LAMP-2A shR) group as compared with control shRNA (CT-shR) group. **b** Activator proteins in apoptosis including caspase 3, caspase 9 and Bax, and anti-apoptotic protein Bcl-2 were measured by western blot. Caspase 3, caspase 9 and Bax expressions were enhanced while Bcl-2 expression was inhibited in LAMP-2A shR group as compared with CT-shR group. **c** TUNEL results of differently treated groups. Significantly more apoptotic cells were found in LAMP-2A shR group as compared with CT-shR group. The data are mean ± SEM from three mice as a group. The levels of *p*-PERK, *p*-IRE1 and *p*-JNK are compared with levels of PERK, IRE1 and JNK respectively, while the rest of the proteins are compared with β-actin. Protein levels are expressed relative to vehicle group set as 1. Significant changes are set as *p* < 0.05 and represented by asterisk (One-Way ANOVA; Bonferroni's test)

To explore the effects of CMA on in vivo glioblastoma growth, we established a xenograft nude mouse model. U87-MG cells were transfected with LAMP-2A shRNA virus to inhibit CMA activation (Fig. 3a). All the experimental mice were at similar healthy status before allocation and no procedure related adverse events were observed. Mice injected with LAMP-2A silenced U87-MG cells showed significantly smaller tumors than those in control shRNA (CT-shRNA) group (Fig. 7a). From post-implantation Day 18, the tumor volume was significantly smaller in mice from LAMP-2A shRNA group than CT-shRNA group, so as the final tumor weight after mice sacrifice (Fig. 7b, c). WB analysis of the tumor tissue demonstrated the decreased LAMP-2A and increased N-CoR expression in LAMP-2A shRNA group. Further IHC study confirmed the resultant accumulation of N-CoR after LAMP-2A being knocked down (Fig. 7e). We further examined activation of UPR after LAMP-2A inhibition, as revealed by significantly enhanced *p*-PERK, *p*-IRE1, *p*-JNK and CHOP expression by WB (Fig. 8a). Moreover, up-regulation of pro-apoptotic proteins and down-regulation of anti-apoptotic proteins were found in mice injected with LAMP-2A deficient U87-MG cells than those in control, which accorded with more apoptotic cells revealed by TUNEL analysis (Fig. 8b, c).

## Discussion

Chaperone-mediated autophagy (CMA) is the first studied autophagy process indicating that lysosome guiding degradation of intracellular proteins can be selective [6]. Since binding of substrates to LAMP-2A is the limiting step, the CMA activity largely depends on the level of LAMP-2A [12]. Enhanced CMA via elevated LAMP-2A expression has been reported in various malignant solid tumors including lung, breast and liver cancers to protect against intracellular stress and promote cancer cell survival [8, 10, 11, 20, 24]. Specific proteins bearing KFERQ-like motif such as ESP8, TP53, N-CoR, hypoxia-inducible factor (HIF-1α) and PED are the downstream targets and mediate the oncogenic effect of CMA [10, 20, 24–28]. Physiologically brain tissue displays low expression of LAMP-2A. Under circumstances of stress and hypoxia, LAMP-2A is significantly elevated, resulting in activated CMA and contributing to cell survival [29]. As for GBM, elevated LAMP-2A was observed in clinical samples [30]. However, the essential function of LAMP-2A, and the involvement of CMA and downstream molecular mechanism in this lethal brain cancer, remain rarely investigated.

In this study, we found up-regulated LAMP-2A and inversely correlated N-CoR expression at protein level in GBM patients. We further discovered suppressed UPR and apoptosis pathway in GBMs with high LAMP-2A level. In vitro study with manipulated LAMP-2A and N-CoR expression demonstrated that CMA mediated N-CoR degradation and relieved UPR and apoptosis. In vivo xenograft model further proved that LAMP-2A silencing inhibited tumor growth and promoted the apoptosis. To the best of our knowledge, this study is the first work to provide evidence of LAMP-2A as potential biomarker and unveil the molecular mechanism of CMA mediated anti-apoptosis effect in GBM. Inhibiting CMA activity by targeting LAMP-2A can be exploited as a potential therapeutic approach in the treatment of malignant glioma.

Recently published research data identified the contribution of GBM cells to the up-regulation of LAMP-2A and CMA activity in pericytes through cell–cell interaction, which ablated pericytes antitumor immune function essential for GBM survival [12]. However, in terms of temozolomide (TMZ) treatment, in vitro studies showed CMA mediated downregulation of HIF-1α improved the response of TMZ-resistant GBM cells, and blocking CMA induced resistance of TMZ-sensitive cells [13, 14]. While in breast cancer inhibiting CMA by downregulating LAMP-2A could augment sensitivity to doxorubicin [11]. Therefore, the role of CMA in GBM tumorigenesis and chemotherapy is more than a unilateral modulation and in-depth research is worthwhile to unveil the complex tumor biology and facilitate the development of anti-GBM strategy.

We demonstrated that the anti-apoptotic effect of CMA in GBM was through degradation of N-CoR and abrogation of downstream UPR. As the core component of a multi-protein repressor complex, N-CoR regulates transcription of various tumor suppressor genes [31]. GBM cell with low or inhibited N-CoR expression showed enhanced invasiveness and increased tumor formation capacity [21, 22]. On the other hand, serine/threonine protein phosphatase 2A (PP2A) inhibitor or combination of retinoic acid and okadaic acid led to disruption of N-CoR complex and its cytoplasmic translocation, and resulted in tumor cell differentiation and death [22, 32, 33]. The phenomenon is attributed to the cytotoxic ER stress UPR induced by uncontrollable cytosolic accumulation of N-CoR [19, 34]. Cancer cells benefit from the loss of function of N-CoR complex and escape from the side effect of N-CoR translocation by means of elevated LAMP-2A and CMA mediated clearance [19, 20].

N-CoR/UPR does not fully account for the protective effect of CMA in GBM, since N-CoR elimination by siRNA could not completely reverse the activation of apoptosis resulted from LAMP-2A inhibition. As a matter of fact, CMA impacts multiple complex pathways to promote cancer survival. Tumor cells induce acetylation of PKM2 by high glucose concentration and deliver it to CMA degradation in order to accumulate glycolytic intermediates for cell growth [28]. CMA is also responsible for degradation of p53, which induces transcriptional down-regulation of various glycolytic enzymes essential for tumor growth [8, 35]. Elevating CMA activity by downregulating autophagy-related gene 5 (ATG5)-dependent macroautophagy stimulates growth and metastasis of breast cancer cells [36]. Similarly, induction of CMA compensates for impaired macroautophagy to promote hepatocellular carcinoma survival [37]. Considering the diverse pathways related to CMA in other cancer types, more researches are worthwhile to depict a complete regulatory network of CMA in GBM development.

## Conclusions

In conclusion, our research proved that LAMP-2A upregulation protected GBM cells from apoptosis by degrading N-CoR and inhibiting downstream UPR. The mechanism of CMA shift between pro-oncogenic effect and chemoresistance to TMZ still remains to be elucidated. Further insights into the interplay between CMA and other biological process essential for GBM development are needed. Last but not least, as a promising biomarker for GBM development, LAMP-2A has remarkable therapeutic implication and effective modulators remain to be investigated.

## Data Availability

The datasets used and/or analysed during the current study are available from the corresponding author on reasonable request.

## Abbreviations

GBM: GlioblastomaGBM
CMA: Chaperone-mediated autophagy
Hsc70: Heat shock cognate 71 kDa protein
LAMP-2A: Lysosomal associated membrane protein 2A
N-CoR: Nuclear receptor co-repressor
UPR: Unfolded protein response
PERK: Pancreatic ER kinase
eIF2α: Eukaryotic translation initiator factor 2α
ATF4: Activating transcription factor 4
CHOP: C/EBP homologous protein
IRE1: Inositol requiring enzyme 1
TRAF2: TNF receptor-associated factor 2
ASK1: Apoptosis signal regulating kinase 1
JNK: JUN N-terminal kinase
LGG: Low grade glioma
PTEZ: Peri-tumor edema zone
qPCR: Quantitative polymerase chain reaction
WB: Western blot
IHC: Immunohistochemistry
DMEM: Dulbecco’s modified Eagle’s medium
FBS: Fetal bovine serum
TUNEL: Terminal deoxynucleotidyl transfer-mediated dUTP nick end labeling
qRT-PCR: Quantitative real-time RT-PCR
HIF-1α: Hypoxia-inducible factor
PP2A: Serine/threonine protein phosphatase 2A
ATG5: Autophagy-related gene 5
Co-IP: Co-immunoprecipitation
CT-siRNA: Control siRNA
CT-shRNA: Control shRNA
